# First Succinylome Profiling of *Vibrio alginolyticus* Reveals Key Role of Lysine Succinylation in Cellular Metabolism and Virulence

**DOI:** 10.3389/fcimb.2020.626574

**Published:** 2021-02-05

**Authors:** Fuyuan Zeng, Huanying Pang, Ying Chen, Hongwei Zheng, Wanxin Li, Srinivasan Ramanathan, Rowena Hoare, Sean J. Monaghan, Xiangmin Lin, Jichang Jian

**Affiliations:** ^1^ Shenzhen Institute, Guangdong Ocean University, Shenzhen, China; ^2^ Fisheries College, Guangdong Ocean University, Zhanjiang, China; ^3^ Guangdong Provincial Key Laboratory of Pathogenic Biology and Epidemiology for Aquatic Economic Animals, Guangdong Key Laboratory of Control for Diseases of Aquatic Economic Animals, Southern Marine Science and Engineering Guangdong Laboratory (Zhan jiang), Zhanjiang, China; ^4^ Key Laboratory of Experimental Marine Biology, Institute of Oceanology, Chinese Academy of Sciences, Qingdao, China, Laboratory for Marine Biology and Biotechnology, Qingdao National Laboratory for Marine Science and Technology, Qingdao, China; ^5^ Fujian Provincial Key Laboratory of Agroecological Processing and Safety Monitoring, School of Life Sciences, Fujian Agriculture and Forestry University, Fuzhou, China; ^6^ Institute of Aquaculture, University of Stirling, Stirling, United Kingdom

**Keywords:** *Vibrio alginolyticus*, lysine succinylation, acetylation, crosstalk, virulence factors

## Abstract

Recent studies have shown that a key strategy of many pathogens is to use post-translational modification (PTMs) to modulate host factors critical for infection. Lysine succinylation (Ksuc) is a major PTM widespread in prokaryotic and eukaryotic cells, and is associated with the regulation of numerous important cellular processes. *Vibrio alginolyticus* is a common pathogen that causes serious disease problems in aquaculture. Here we used the affinity enrichment method with LC-MS/MS to report the first identification of 2082 lysine succinylation sites on 671 proteins in *V. alginolyticus*, and compared this with the lysine acetylation of *V. alginolyticus* in our previous work. The Ksuc modification of SodB and PEPCK proteins were further validated by Co-immunoprecipitation combined with Western blotting. Bioinformatics analysis showed that the identified lysine succinylated proteins are involved in various biological processes and central metabolism pathways. Moreover, a total of 1,005 (25.4%) succinyl sites on 502 (37.3%) proteins were also found to be acetylated, which indicated that an extensive crosstalk between acetylation and succinylation in *V. alginolyticus* occurs, especially in three central metabolic pathways: glycolysis/gluconeogenesis, TCA cycle, and pyruvate metabolism. Furthermore, we found at least 50 (7.45%) succinylated virulence factors, including LuxS, Tdh, SodB, PEPCK, ClpP, and the Sec system to play an important role in bacterial virulence. Taken together, this systematic analysis provides a basis for further study on the pathophysiological role of lysine succinylation in *V. alginolyticus* and provides targets for the development of attenuated vaccines.

## Introduction

Protein post-translational modifications (PTMs) are vital regulatory mechanisms, which are involved in a plethora of cellular events such as gene expression, virulence, and cellular metabolism in both prokaryotic and eukaryotic cells (Avison et al., 2002; Xie et al., 2014). During these processes simple chemical groups such as a methyl, hydroxyl, phosphate, and acetyl groups or more complex groups such as sugars, lipids, AMP, and ADP-ribose may be added to the protein molecules (Ribet and Cossart, 2010). Several types of PTMs have been discovered that are involved in bacterial virulence and physiology. Hence, determining bacterial proteomes alone may be limiting and characterization of PTMs is vital to better understand adaption, virulence, and resistance of bacterial pathogens (Wu et al., 2019). Among the 20 amino acids residues, lysine is frequently targeted for a variety of PTMs, for example the protein Nϵ-acylation targets lysine residues and is an extensively dispersed PTM (Komine-Abe et al., 2017). Recent research has consistently revealed that lysine can be post-translationally modified by numerous types of acylation (Weinert et al., 2013). Among the hundreds of diverse PTMs, acylation on lysine residues, such as lysine crotonylation (K_cr_), lysine propionylation (K_pr_), lysine glutarylation (K_glu_), lysine butyrylation (K_bu_), lysine malonylation (K_mal_), lysine β-hydroxybutyrylation (K_bhb_), and lysine 2-hydroxyisobutyrylation (K_hib_) are vital for efficient regulation of many prokaryotic and eukaryotic proteins (Yang et al., 2015).

Protein lysine succinylation (K_suc_), also referred to as Nε-succinylation, is a newly identified and evolutionarily conserved reversible PTM from prokaryotes to eukaryotes. It transfers the succinyl group (-CO-CH_2_-CH_2_-CO-) from the succinyl-CoA to the lysine residue of the protein moiety, resulting in the formation of succinyl-lysine (Zhang et al., 2011). Recently, numerous lysine-succinylated proteins have been identified in various bacterial pathogens, such as *Mycobacterium tuberculosis*, *Porphyromonas gingivalis*, *Candida albicans* (Yang et al., 2015; Zheng et al., 2016; Wu et al., 2019), and so on. Many are enzymes involved in various metabolic pathways and regulation of several central metabolic processes in the bacteria such as glycolysis, gluconeogenesis, the tricarboxylic acid cycle (TCA cycle), and fatty acid metabolism (Xie et al., 2014). Furthermore, Nε-succinylation has been reported in many protein substrates and involved in the regulation of cellular physiology and metabolism in both prokaryotic and eukaryotic cells (Nadine et al., 2017). This PTM can make prominent modifications in structure regulation and protein function. The identification of protein succinylation sites has important implications with regards to understanding of cellular physiology and pathology, potentially leading to valuable information for drug development and biomedical research. In recent times, high-throughput approaches in conjunction with mass spectrometry have been widely applied to identify the K_suc_ in several organisms ranging from bacteria to humans (Colak et al., 2013; Li et al., 2014; Jin and Wu, 2016; Xu et al., 2016; Feng et al., 2017; Song et al., 2017; Xie et al., 2017; Zhang et al., 2017).

*Vibrio alginolyticus* is a Gram-negative halophilic bacterium and an etiological agent of vibriosis, mainly found in marine and estuarine environments. Outbreaks cause high mortality in marine animals with serious economic losses worldwide. Being a zoonotic pathogen, it not only causes vibriosis in marine animals, but also causes foodborne related infections in humans by consumption of contaminated raw and half-cooked seafood (Dan et al., 2019; Zheng et al., 2019). Moreover, several researchers frequently reported antibiotic resistant strains of the bacterium from aquaculture and clinical settings (Horii et al., 2005; Ferrini et al., 2008; Xiong et al., 2010). *V. alginolyticus* is able to form biofilms and is capable of flagellar mediated motility (Echazarreta and Klose, 2019). It also secretes several virulence factors such as, serine protease, hemolysin, exopolysaccharide, siderophores, and cell surface hydrophobicity products through various metabolic pathways (Yang et al., 2015; Hernández-Robles et al., 2016; Santhakumari et al., 2017; Huang et al., 2018), which all contribute to mechanisms of pathogenicity that require further understanding.

Comprehensive lysine succinylome studies conducted in different bacterial pathogens have revealed the importance of this PTM. However, to the best of our knowledge, no succinylated proteins have been discovered so far in *V. alginolyticus*, which presents a foremost obstacle for understanding the regulatory mechanism of K_suc_ in this pathogen. We have therefore conducted the first systematic analysis to identify the targets of this K_suc_ in *V. alginolyticus*. Following enrichment of succinylated peptides from digested cell lysates we used mass spectrometry to explore Nε-succinylation PTMs and identified 2082 K_suc_ sites on 671 proteins in *V. alginolyticus*. Further, the bioinformatic analysis showed that a large quantity of the succinylation sites were present on proteins associated with metabolism pathway, followed by biosynthesis of antibiotics, but also associated with diverse biological processes and functions, such as ribosomes, biosynthesis of secondary metabolites. The results obtained provide the first global lysine succinylation profiling of *V. alginolyticus* and sets a foundation for further investigations on the biological role of lysine succinylation in this bacterial pathogen.

## Materials and Methods

### Bacterial Strains and Sample Preparation

*V. alginolyticus* strain HY9901 was isolated from diseased fish *Lutjanus erythopterus* in Zhanjiang harbor area of Guangdong Province (Cai et al., 2007), China, and cultured in Dulbecco’s Modified Eagle Medium (DMEM) media. The strain was grown overnight in DMEM media, and culture was diluted 1:100 ratio in the fresh DMEM media. Cell were harvested when OD600nm reached 1.0, centrifuged at 8,000×g, and then washed twice with phosphate buffered saline (PBS, NaCl 136.89 mM, KCl 2.67 mM, Na_2_HPO_4_ 8.1 mM, KH_2_PO_4_ 1.76 mM, pH 7.4). The pellets were resolved in 8 M urea and 0.2% SDS in 50 mM Tris-HCl buffer (pH 8.0) and cells were broken by super-sonication on ice for a total of 10 min with 9 s intervals, and the lysate centrifuged at 12,000×g for 15 min at 4°C. The supernatant dithiothreitol (DTT) was added until a final concentration of 2 mM DTT was obtained. The sample was then incubated at 56°C for 1 h, and then the equivalent of 4× the sample volume of pre-cooled acetone was added to precipitate proteins at −20°C for >2 h. The sample pellet was washed twice by centrifugation with pre-cooled acetone. Finally the pellet was dissolved in dissolution buffer containing 0.1 M triethylammonium bicarbonate (TEAB, pH 8.5) and 8 M urea. Protein concentration was determined with a Bradford assay (He et al., 2016).

### Enrichment of Lysine-Succinylated Peptides

Approximately 10 mg of protein sample was used for reduction and alkylation with 10 mM DTT and 20 mM iodoacetamide (IAA), respectively, as described previously (Yao et al., 2019). The treated sample was digested to peptides using trypsin at 1:20 ratio (m/v) at 37°C for 16 h. The lysine-succinylated peptides were enriched by immunoaffinity using agarose-conjugated anti-succinyllysine antibody (PTM Biolabs Inc., Hangzhou, China), as previously described (Yang et al., 2015). Briefly, the digested peptides were incubated with anti-succinyllysine agarose beads overnight at 4°C in NETN buffer (100 mM NaCl, 50 mM Tris-HCl, 1 mM EDTA, and 0.5% (v/v) Nonidet P-40, pH 8.0). The enriched peptides were eluted with 1% trifluoroacetic acid (TFA) and desalted with C18 ZipTips (Millipore, Burlington, MA, USA) before being subjected to MS identification.

### LC-MS/MS Analysis

Proteomic analyses were performed using an EASY-nLC™ 1200 UHPLC system (ThermoFisher Scientific, Germany) coupled to an Orbitrap Q Exactive HF-X mass spectrometer (ThermoFisher) operating in the data-dependent acquisition (DDA) mode which was carried out as previously described (Pang et al., 2020).

### Data Processing

The resulting MS raw data were processed using Proteome Discoverer2.2 software for database retrieval and protein quantification. Tandem mass spectra were compared against the Uniprot_*Vibrio alginolyticus* protein database (4,338 sequences). Trypsin was specified as a cleavage enzyme allowing up to two missing cleavages. The precursor and fragment ion mass tolerance were set to 10 ppm and 0.02 Da. Carbamidomethylation on Cys was specified as a fixed modification and succinylation on protein N-terminals were specified as variable modifications. False discovery rate (FDR) thresholds for peptide and protein were specified at 0.05. Minimum peptide length was set at 7. Lysine succinylation sites were identified with a localization probability set as >0.75. The mass spectrometry proteomics data have been deposited to the ProteomeXchange Consortium (http://proteomecentral.proteomexchange.org) *via* the iProX partner repository with the dataset identifier PXD023153.

### Co-Immunoprecipitation and Western Blotting

Specific polyclonal antibodies to SodB and PEPCK (Phosphoenolpyruvate carboxykinase, one of the key enzymes in gluconeogenesis pathway) were used to precipitate target proteins. *V. alginolyticus* strain cell lysates (500 µg) were interacted with SodB and PEPCK antibody at 4°C overnight. Protein A/G beads washed three times with PBS buffer were added to the lysates at 4°C for 1–3 h (Cheng et al., 2019). The beads were pelleted at 4°C, followed by five washes with PBS buffer. Then 50 μl of loading sample buffer (250 mM Tric-HCl pH = 6.8, 10% SDS, 0.5% bromophenol blue, 50% glycerol, and 5% β-mercaptoethanol) was added to the pellet, boiled for 5 min, and subsequently analyzed by SDS-PAGE and Western blotting.

For Western blotting, proteins were run on 12% 1-DE gels and transferred to a polyvinylidene fluoride (PVDF, Millipore, Billerica, MA, USA) membrane. The membranes were blocked in Tris buffered saline (TBS, 500 mM Tris-HCl; 2.8 M NaCl; 60 mM KCl; pH7.4) containing 0.05% (v/v) Tween 20 with 5% (w/v) skimmed milk and incubated for 1 h at room temperature. The primary antibodies used in the western blot were anti-SodB (1:4,000), anti-PEPCK (1:4,000), and anti-succinyllysine mouse mAb (PTM Biolabs Inc., Hangzhou, China) (1:5m000 in TBST with 5% skimmed milk) and incubated overnight at 4°C. Horseradish peroxidase (HRP) conjugated goat anti-mouse IgG (H+L) (Beyotime Biotechnology, Shanghai, China) was used as the secondary antibody at a 1:10,000 dilution in TBST with 3% skimmed milk. Finally, the membrane was visualized using the ECL system (Bio-Rad, Hercules, CA, USA), and recorded by the ChemiDoc™ MP (Bio-Rad, Hercules, CA, USA) imaging system (Wang et al., 2019).

### Bioinformatics

Gene Ontology (GO, including cellular components, molecular functions, and biological processes) and the Kyoto Encyclopedia of Genes and Genomes (KEGG) pathway annotation of identified succinylated proteins were performed using online software OmicsBean (http://www.omicsbean.cn/). The Cluster of Orthologous Groups of proteins (COG) was analyzed using the COG database of NCBI (https://www.ncbi.nlm.nih.gov/COG/). STRING software (version 11.0) was used to annotate protein domains. Amino acid sequence motifs were analyzed using online software MoMo (Modification Motifs, version 5.1.1, http://meme-suite.org/tools/momo?tdsourcetag=s_pcqq_aiomsg) (Cheng et al., 2017). All analyses with a corrected p-value <0.05 were considered significant, and using GraphPad Prism 8.0 software to generate images. Protein-Protein interactions (PPIs) were predicted using STRING (https://string-db.org/) combined with Cyctoscape 3.7.1 software.

## Results and Discussion

### Identification of Lysine-Succinylated Peptides and Proteins in *V. alginolyticus*

**Figure 1:**
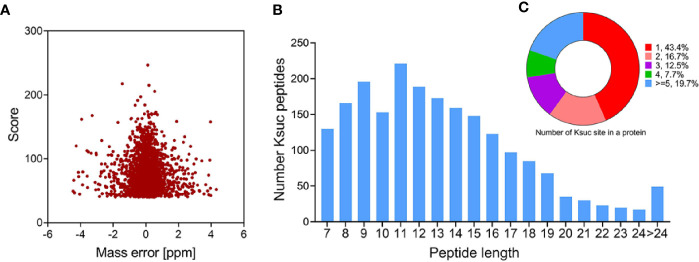
Profile of *V. alginolyticus* lysine succinylation proteome. **(A)** Distributions of mass errors for lysine succinylated peptides. **(B)** Distribution of lysine succinylated peptides based on their length. **(C)** The pie chart shows the distribution of succinylation sites in each protein.

We combined immunoaffinity enrichment of lysine-succinylated peptides with a highly specific succinylation antibody and LC-MS/MS to profile the succinylated proteins and peptides of *V. alginolyticus*. With FDR thresholds below 5% for peptides, 2,082 unique succinylated peptides with 2,082 succinylation sites from 671 proteins were identified in *V. alginolyticus* (**Supplemental Tables S1**, **S2**). The mass error of succinylated peptides ranged from −5 to 5 ppm, illustrating that the MS dataset was controlled within an expected error rate (**Figure 1A**). The peptides exhibit distinct abundance depending on their lengths, and most were in range of 7–24 segments (97.65%), with a small number of peptides with lengths of 24–38, which accounted for about 2.35% (**Figure 1B**). Moreover, of the 671 succinylated proteins, 43.4% were succinylated at a sole site, 16.7, 12.5, and 7.7% were modified at two, three, and four sites, respectively, whereas 19.7% were modified at five or more sites (**Figure 1C**). In *V. alginolyticus* the most heavily succinylated protein was DNA-directed RNA polymerase subunit beta RpoS (28 sites). In addition, nine proteins exhibited high abundances (>15) of succinylated sites including translation process related proteins D0WXZ6 (RpsA, 19 sites), D0WW35 (RpoC, 18), D0WYY9 (FusA, 17), and D0WX73 (Frr, 16); the major chaperone proteins D0WYT6 (GroL, 18) and D0WUB9 (DnaK, 16); pyruvate dehydrogenase E1 component D0WZ79 (18); dihydrolipoyl dehydrogenase D0WZ77 (LpdA, 16); AAA_PrkA domain-containing protein A0A2I3BY81 (16). The abundance of lysine succinylation sites in chaperone proteins found in this study, which are consistent with the results of pathogenic bacteria such as *Aeromonas hydrophila* and *M. tuberculosis*, is worthy of further investigation (Xie et al., 2014; Yao et al., 2019).

### Functional Annotation of the Lysine Succinylome in *V. alginolyticus*

**Figure 2:**
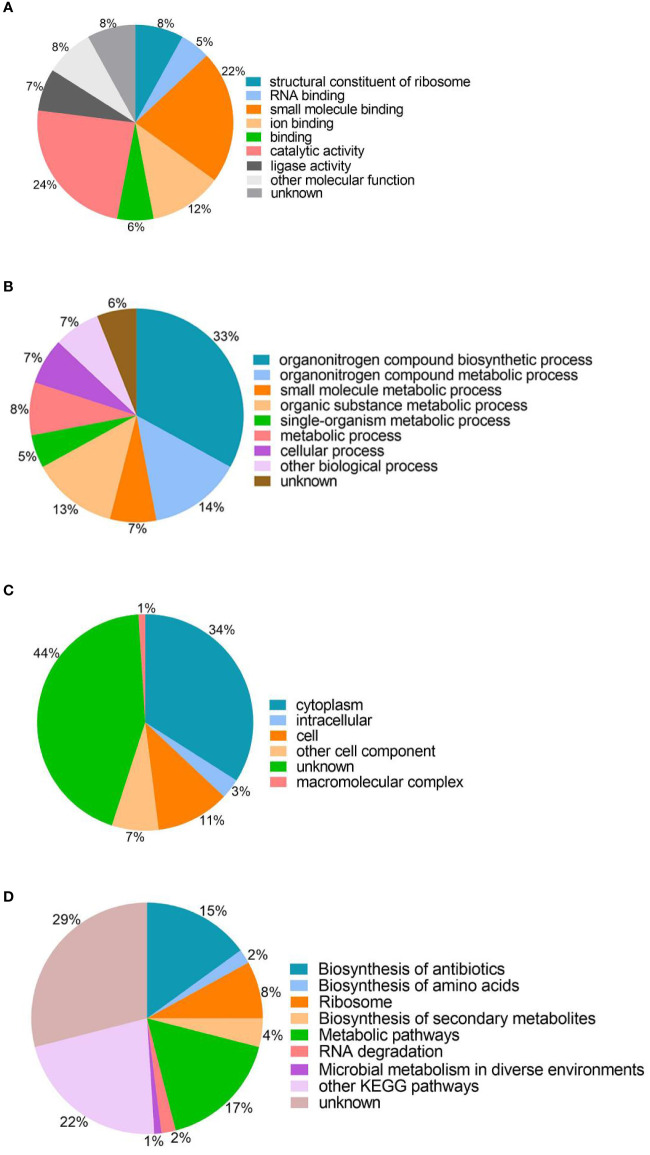
Gene ontology functional classification and KEGG pathway analysis of the identified succinylated proteins. **(A)** Molecular function. **(B)** Biological processes. **(C)** Cell components. **(D)** KEGG pathway analysis.

To understand the roles of lysine succinylation, we performed GO, KEGG, COG, and domain analysis of all identified succinylated proteins. The classification results relating to molecular function, biological process, and cellular component categories showed that the largest protein group of succinyl proteins are associated with catalytic activity, organonitrogen compound biosynthetic processes, and cytoplasm, which accounts for 24, 33, and 34% of the total succinyl proteins, respectively (**Figure 2**). Moreover, other molecular functions include small molecule binding, ion binding, and structural constituents of ribosomes, representing 22, 12, and 8% of all identified proteins, respectively (**Figure 2A**). The other large groups in terms of biological processes are proteins associated with organonitrogen compound metabolic processes (14%), organic substance metabolic processes (13%), and metabolic processes (8%) (**Figure 2B**). Cell (11%), other cell components (7%), intracellular (3%), and macromolecular complexes (1%) are classified in cellular components (**Figure 2C**). The GO analysis of the succinylome suggests that the succinylated proteins are related to different molecular functions, biological processes, and cellular components, and closely related to bacterial life activities.

The KEGG analysis of the succinylated proteins showed that most identified proteins were enriched in metabolic pathways (17%), biosynthesis of antibiotics (15%), ribosomes (8%), and 29% succinylated protein were not enriched in the metabolic pathway category (**Figure 2D**). In this study, we found that 54 ribosomal proteins were succinylated, including 21 30S ribosomal proteins and 33 50S ribosomal proteins were related to translation processes. Interestingly, succinylation of ribosomal proteins was also found in *M. tuberculosis*, *A. hydrophila*, and *E. coli* (Colak et al., 2013; Xie et al., 2014; Yao et al., 2019).

**Figure 3:**
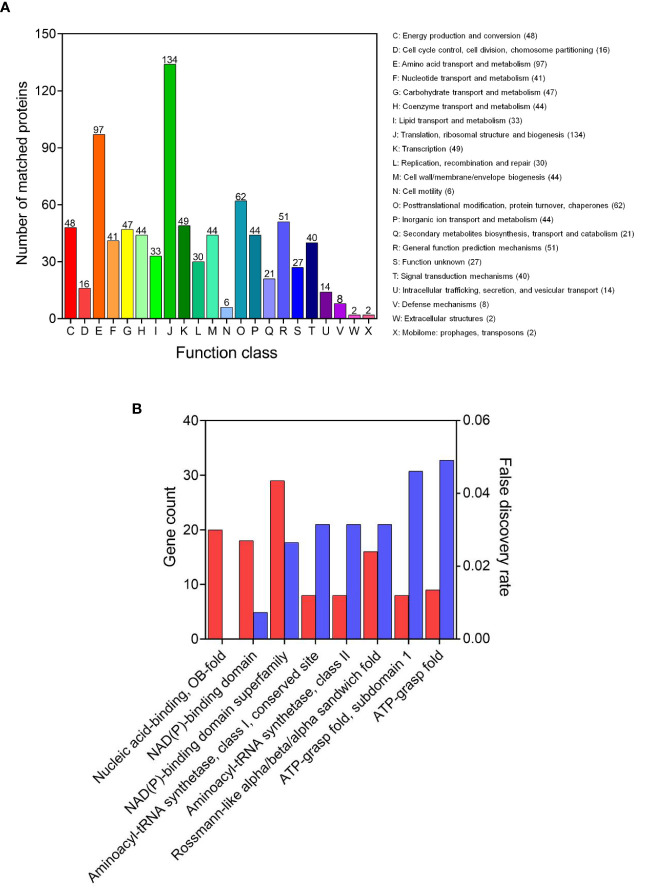
Functional annotation of the lysine succinylome in *V. alginolyticus*. **(A)** The COG analysis and **(B)** domains enrichment analysis of the succinylated proteins.

The COG is a tool for genome-scale analysis of protein functions and evolution. In this study, COG analysis revealed that translation, ribosomal structure and biogenesis (134 succinylated proteins), amino acid transport and metabolism (97), posttranslational modification, protein turnover, chaperones (62), and general function prediction mechanisms (51), were significant (**Figure 3A**). Our results were consistent with previous succinyl-proteome studies conducted in *E. coli*, *M. tuberculosis*, *B. subtilis*, and *V. parahaemolyticus* (Colak et al., 2013; Kosono et al., 2015; Pan et al., 2015; Yang et al., 2015), which revealed that the majority of succinyl-proteins consisted of translation and metabolic proteins.

The domain is the structural basis of protein physiological function, thus in order to further identify the function associated with succinylation, the domain of the identified succinylated proteins were annotated. The results shown in **Figure 3B**, indicate enriched succinylated substrates with functional domains including NAD(P)-binding domain superfamily, nucleic acid-binding, OB-fold, NAD(P)-binding domain, and Rossmann-like alpha/beta/alpha sandwich fold, were the largest. In addition, 16 aminoacyl-tRNA synthetases and 9 ATP dependent proteases underwent succinylation, suggesting that succinylation modification may be involved in the protein synthesis and regulation of ATPase activity, which is consistent with the succinylation observed in the fish pathogen *A. hydrophila* (Yao et al., 2019).

### Motif of Succinylated Peptides in *V. alginolyticus*

**Figure 4:**
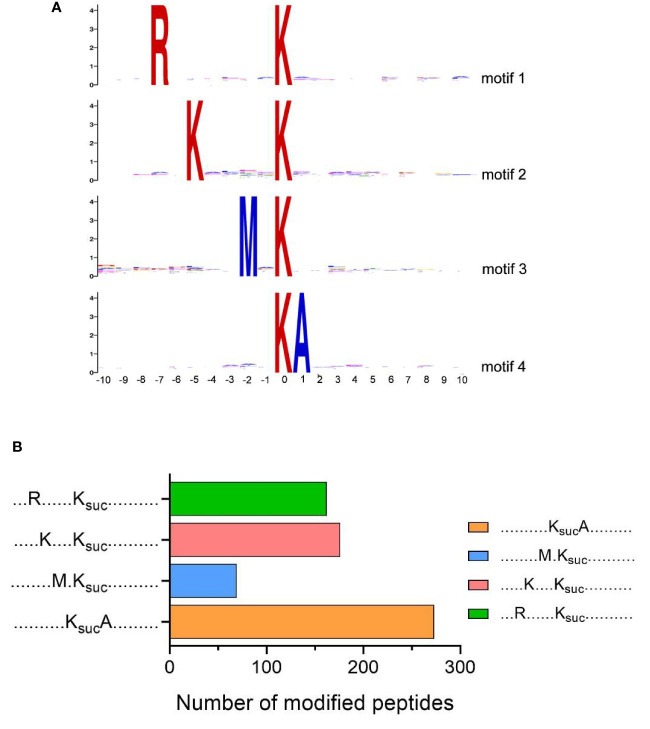
Motif analysis of lysine succinylation sites. **(A)** Sequence logos of motifs (*P*-value <0.000001) identified by MoMo software. **(B)** Numbers of each identified motifs.

We further evaluated the position-specific amino acid of succinylated peptides, using MoMo software to analyze the surrounding sequences (10 amino acids to both termini) of succinylated lysine sites in the *V. alginolyticus* succinyl-proteome (p-value <0.000001). The results showed that four conserved motifs were significantly over-represented around the lysine succinylation sites, which tended to have arginine (R) at position -7, lysine (K) at position -5, methionine (M) at position -2, and alanine (A) at position +1 (**Figure 4A**). The frequency of K_suc_A motif was the highest, K_(-5)_K_suc_ and R_(-7)_K_suc_ motif the second highest, and M_(-2)_K_suc_ motif the lowest (**Figure 4B**). Similar results were observed for the succinylome of *Deinococcus radiodurans* (K_(-5)_K_suc_ motif), *V. parahaemolyticus* and rice leaves (R_(-7)_K_suc_ motif), which suggests bacteria and plants may share common conserved motifs surrounding lysine succinylated sites (Pan et al., 2015; Zhou et al., 2018; Zhou et al., 2019). Then, when we compared our motifs to the reported succinylome of fish pathogens *A. hydrophila* and *V. parahaemolyticus*, the result found conserved motifs in arginine (R) and lysine (K), although the precise positions varied (Pan et al., 2015; Yao et al., 2019). Furthermore, we also found that the preference for alanine (A) at position +1 (KsucA motif) is a unique feature of a succinylated modified protein in *V. alginolyticus*.

### Validation of SodB and PEPCK Lysine-Succinylated Proteins Using Co-Immunoprecipitation and Western Blotting

**Figure 5:**
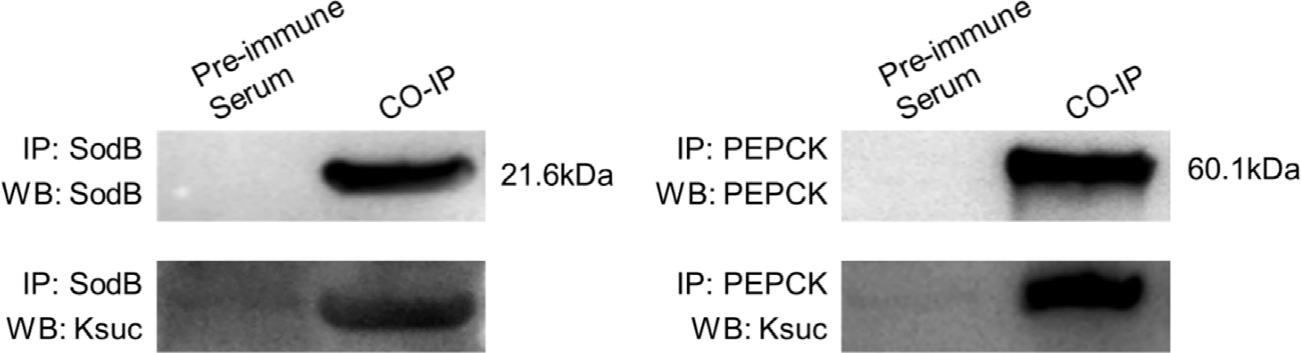
Validation of SodB and PEPCK lysine-succinylated proteins in *V. alginolyticus* using Co-Immunoprecipitation and Western blotting. SodB and PEPCK proteins were enriched by Co-IP with specific antibodies, followed by Western blotting with SodB and PEPCK proteins specific antibodies (above), and Western blotting with anti-lysine succinylation antibodies (below).

To further validate the identified lysine-succinylated results, two Ksuc proteins (SodB and PEPCK) were selected and analyzed by Co-IP and Western blotting. The SodB and PEPCK proteins were captured by their respective antibodies and then Western blotting was performed with anti-succinylation and anti-target protein antibody, respectively (**Figure 5**). The results showed that SodB and PEPCK proteins exhibited succinylation modifications consistent with lysine-succinylated proteomic data, further validating our proteomics results.

### Overlap Between Lysine Succinylation and Acetylation in *V. alginolyticus*

**Figure 6:**
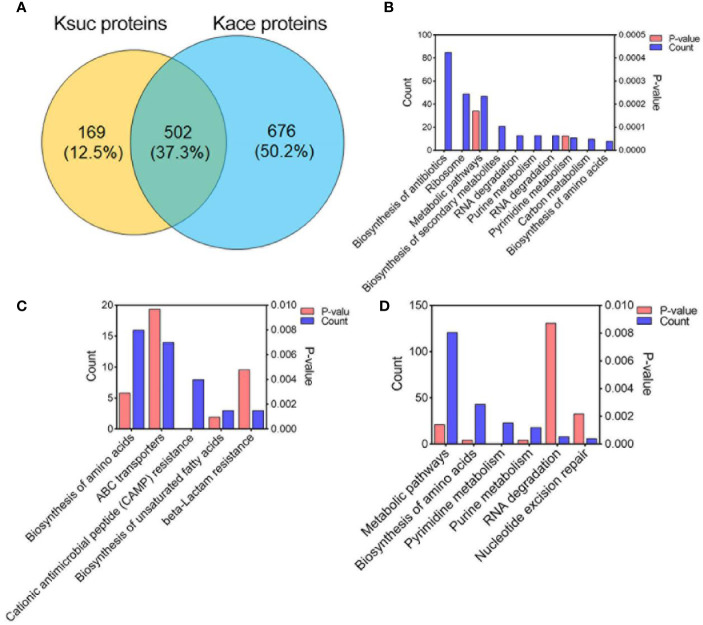
Comparison of succinylated and acetylated proteins in *V. alginolyticus*. **(A)** Overlap between succinylated and acetylated proteins in *V. alginolyticus*. **(B–D)** KEGG pathway enrichment analysis of the overlapped proteins, specific succinylated modified proteins, and specific acetylated modified proteins.

**Figure 7:**
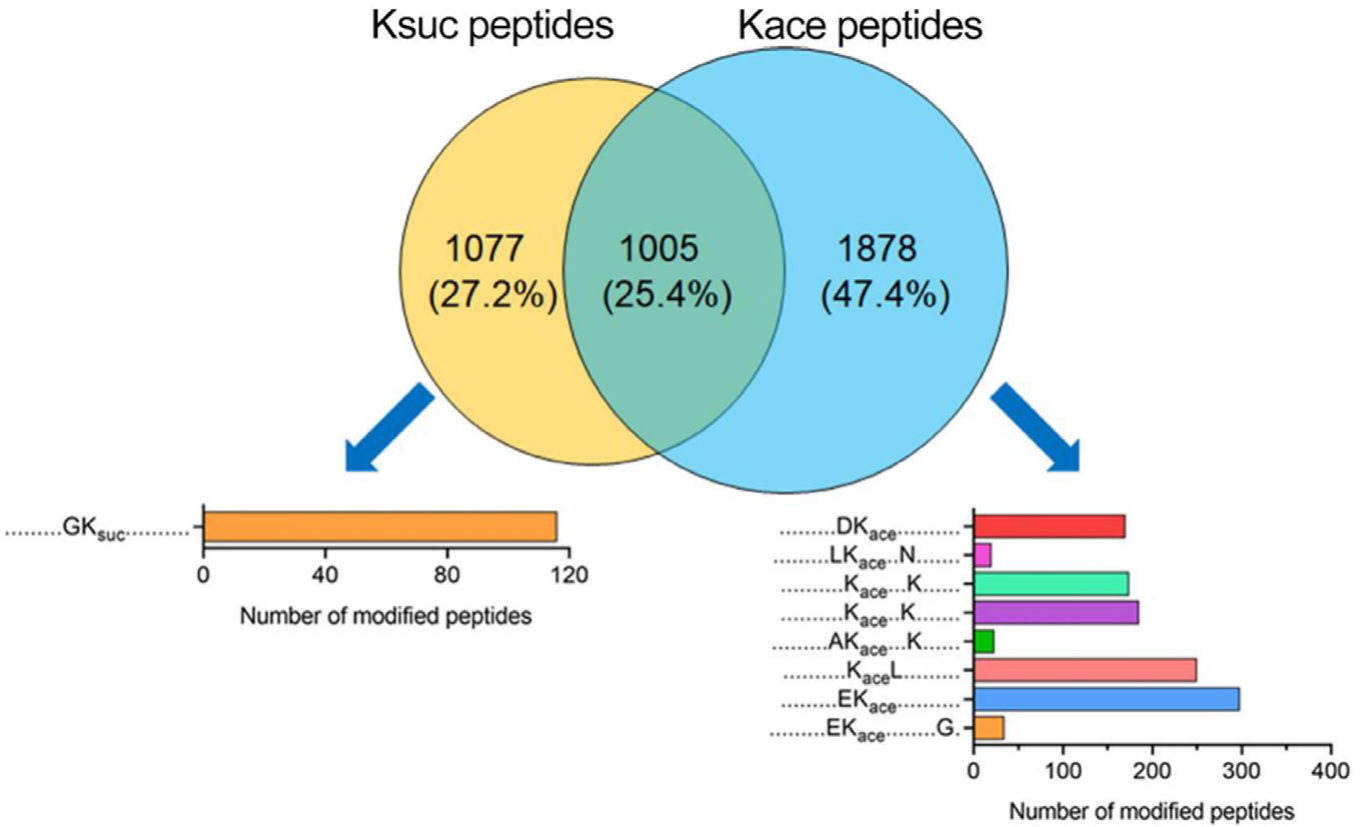
Comparison of succinylation and acetylation peptides in *V. alginolyticus* Overlap between succinylated and acetylated peptides in *V. alginolyticus*, and motif analysis.

Previous studies have shown that there are various modifications in lysine residues, such as acetylation, succinylation, propionylation, formylation, ubiquitination (Yang and Seto, 2008). In our previous report on the acetylome of *V. alginolyticus* we identified 2,883 acetylated sites within 1,178 proteins. In order to determine whether succinylation and acetylation “crosstalk” occurs at the same lysine site, we compared the lysine succinylation data here to the previous acetylation data on post-translationally modified proteins and peptides (**Figures 6** and **7**). The comparison results showed that 502 proteins overlapped (**Figure 6A**), and further enrichment analysis of KEGG pathways was performed. Of the overlapped proteins, a total of 10 KEGG pathways are enriched, of which biosynthesis of antibiotics, ribosome, and metabolic pathways are dominant (**Figure 6B**). Among the 169 specific succinylated modified proteins alone, five KEGG pathways were enriched, mainly amino acid biosynthesis, ABC transporters, and cationic antimicrobial peptide (CAMP) resistance (**Figure 6C**), while in 676 specific acetylated modified proteins, were enriched in six KEGG pathways of which metabolic pathways, biosynthesis of amino acids and pyrimidine metabolism are predominant (**Figure 6D**). Further analysis showed that DNA binding protein RpoB included 28 K_suc_ sites and 6 K_ace_ sites, while fatty acid oxidation complex subunit alpha YfcX included 2 K_suc_ sites and 17 K_ace_ sites, suggested that there is a significant difference in the number of acetylation and succinylation sites of the same modified protein.

At the peptide level, 1,005 peptides were overlapped, and 1,077 K_suc_-specific peptides, and 1,878 K_ace_-specific peptides were identified (**Figure 7**). The conserved motif analysis showed that the overlapped peptides were not enriched, but in K_suc_-specific peptides one conserved motif was enriched in (GK_suc_ motif), and in K_ace_-specific peptides eight conserved motifs were enriched including DK_ace_, LK_ace_N(+3), K_ace_K(+4), K_ace_K(+3), AK_ace_K(+4), K_ace_L, EK_ace_, EK_ace_G(+9) motif, and EK_ace_ motif with the greatest enrichment.

**Figure 8:**
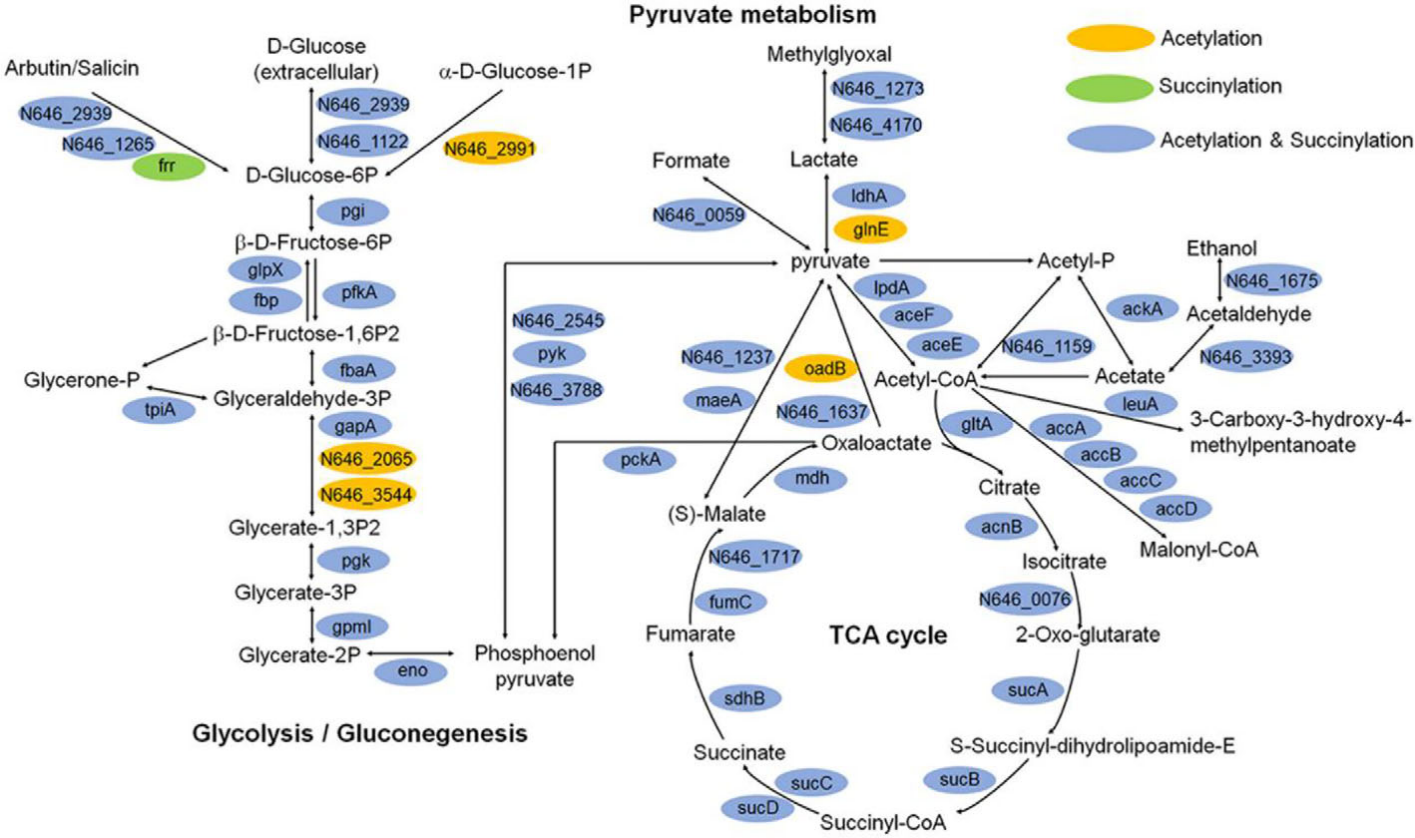
Key enzymes with succinylation and acetylation modification in glycolysis/gluconeogenesis, TCA cycle, and pyruvate metabolism pathways.

Moreover, we illustrated the occurrence of lysine succinylation and acetylation in three central metabolic pathways: the glycolysis/gluconeogenesis, TCA cycle, and pyruvate metabolism. The results indicated that the enzymes in three pathways were acetylated (except for *frr* gene) and the majority of the enzymes were also found to be succinylated (except for *N646_2991*, *N646_2065*, *N646_3544*, *glnE*, and *oadB* genes) (**Figure 8**), our results are similar with those obtained in others bacteria, such as *V. alginolyticus*, *A. hydrophila*, *Pseudomonas aeruginos*a (Pan et al., 2015; Gaviard et al., 2018; Sun et al., 2019). These results together reflect that the two types of lysine modification are highly enriched and widely overlapped in metabolism and ribosome related proteins (**Figures 6B** and **8**), suggesting that both types of modifications may play an important role in the regulation of cellular processes, especially in central metabolism and ribosome activity.

### Virulence Factors of Succinylated Proteins in *V. alginolyticus*

**Figure 9:**
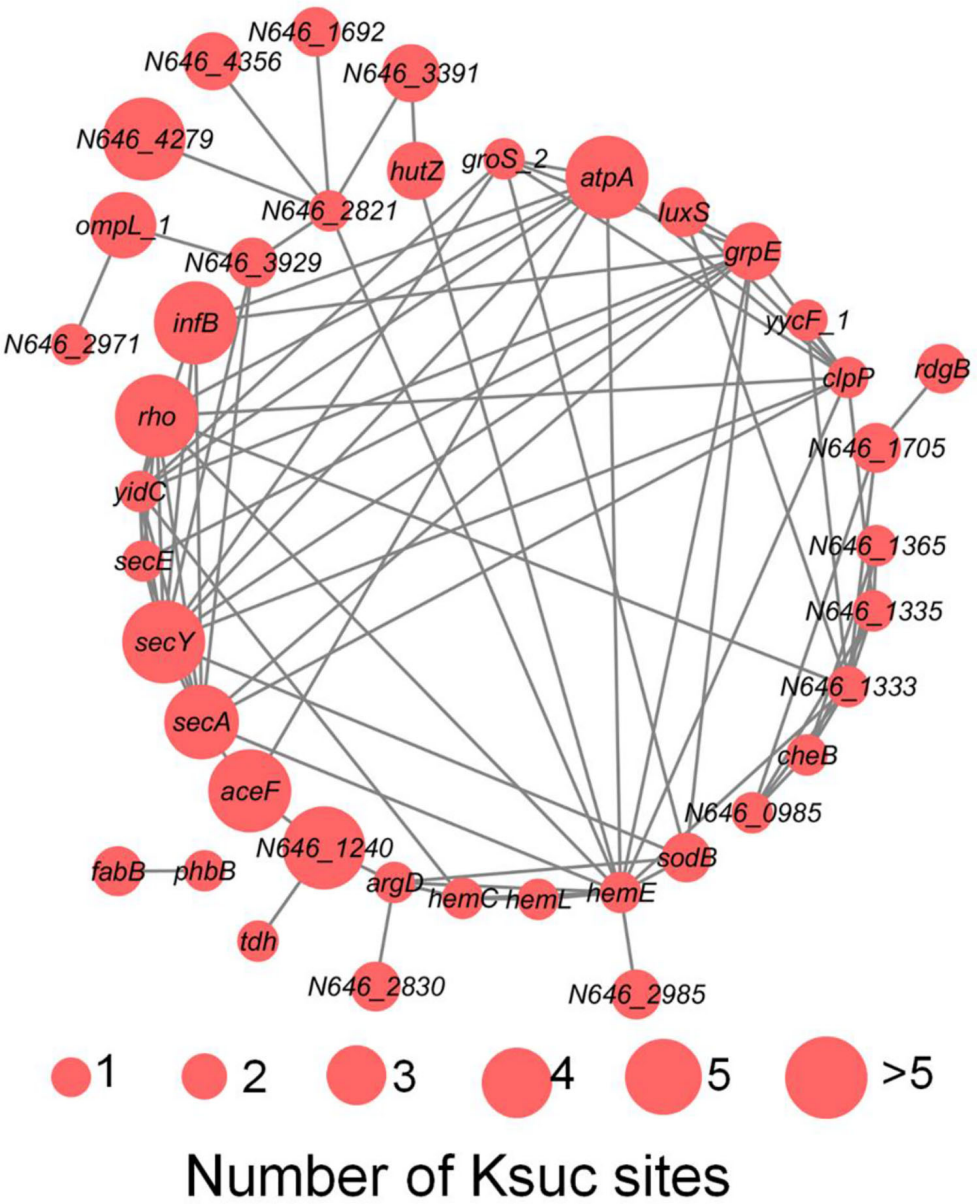
The PPI network of succinylated virulence factors in *V. alginolyticus*. The size of the circle represents the number of Ksuc sites.

*V. alginolyticus* is an important pathogen in aquaculture, which infects a variety of fish, shrimp and shellfish leading to great economic losses around the world, and also contributes to disease in humans inducing symptoms such as fever, nausea, diarrhea, and extra intestinal infections (Sasikala and Srinivasan, 2016; Yu et al., 2019). Previously, it has been reported that protein post-translational modification is closely related to bacterial virulence, such as acetylation, succinylation, and phosphorylation (Whitmore and Lamont, 2012; Ren et al., 2017; Gaviard et al., 2019). Virulence factors (VFs) are the basis of pathogenicity of *V. alginolyticus*, so it is of great significance to study VFs. In this study, using online VFDB software analysis, we detected a total of 50 (7.45% of total K_suc_ proteins) succinylated VFs in *V. alginolyticus*, and the protein-protein interaction network among the 50 VFs proteins using online STRING database combined with Cytoscape software, showed that 40 proteins were found to interact (**Figure 9**). The results showed that VFs were enriched in the following pathways, including bacterial chemotaxis, the bacterial secretion system, TonB-dependent receptor family, and microbial metabolism in different environments, and previous reports have shown that these pathways are closely related to bacterial virulence (Wang et al., 2008; Goldberg et al., 2010; Kapitein and Mogk, 2013; Erhardt, 2016; Green and Mecsas, 2016; Guo et al., 2017).

**Figure 9** showed that S-ribosylhomocystein lyase (LuxS) is a key enzyme in quorum sensing and has two Ksuc sites. Previous studies have found that this enzyme plays an important role in virulence (Coulthurst et al., 2004), and in *A. hydrophila* research it has been reported that LuxS exhibits cross-talk between lysine acetylation and succinylation, and Yao et al. research showed that the succinylation of lysines on LuxS at the K23 and K30 sites positively regulate the production of the quorum sensing autoinducer AI-2, and that these PTMs ultimately alter its competitiveness with *V. alginolyticus* (Sun et al., 2019; Yao et al., 2019). This study showed the succinylation of lysines on LuxS at the K29 and K45 sites. But whether they have the same function as LuxS in *A. hydrophila* remains to be further studied in *V. alginolyticus*.

In Gram-negative bacteria, the Sec system can transport a variety of proteins into the extracellular medium, including toxins and enzymes (Chatzi et al., 2013), which play an important role in bacterial virulence. In the Sec system SecA plays a central role in directing Sec-dependent transport, while SecE and SecY are membrane proteins that form a channel in the membrane which provides the core molecular machinery to direct secretion (Crane and Randall, 2017). Another Sec protein, YidC, is very important to *E. coli* survival and deletion of YidC will interfere with the insertion of Sec-dependent membrane proteins, thus affecting secretion processes of bacteria (Samuelson et al., 2000). In this study, we found that SecAEY and YidC proteins were succinylated, and SecA and SecY had five and six modification sites, respectively, indicating that succinylation modification plays a central role in the Sec system.

Other virulence factors such as *tdh*, *sodB*, *clpP* were also succinylated. *tdh* gene encodes thermostable direct hemolysin (TDH), which is a major virulence factor in *V. alginolytic*us (Avsever, 2016). Our previous research found that *sodb* and *clpp* genes are important virulence factors of *V. alginolyticus*, and deletion of those genes leads to reduction of bacterial virulence, suggesting they have potential application for the construction of live attenuated vaccines (Chen et al., 2019a; Chen et al., 2019b). Many virulence factors of *V. alginolyticus* were found to be succinylated in this study, indicating that lysine succinylation may play a crucial role in regulating the virulence of *V. alginolyticus*.

## Conclusion

*Vibrio alginolyticus* is an opportunistic and halophilic Gram-negative pathogen, which impedes development of the aquaculture sector for some species of fish and affects human health. However, the intrinsic biological behavior of *V. alginolyticus* is largely unknown. Many studies have shown that succinylation of lysine within proteins is involved in the regulation of bacterial physiology and plays a major role in many biological processes. In this study we successfully identified a total of 2,082 succinylation sites matched with 671 proteins in *V. alginolyticus*. Of these 1,005 peptides and 502 proteins overlapped with acetylated proteins, indicating extensive overlap between these two PTMs, and these proteins were involved in glycolysis/gluconeogenesis, TCA cycle, and pyruvate metabolism. In conclusion, the succinylome of *V. alginolyticus* was analyzed for the first time revealing possible biological roles of lysine succinylated proteins, 7.5% of which were predicted to be virulence factors and may thus provide possible targets for the development of attenuated vaccines.

## Data Availability Statement

The mass spectrometry proteomics data have been deposited to the ProteomeXchange Consortium (http://proteomecentral.proteomexchange.org) *via* the iProX partner repository with the dataset identifier PXD023153.

## Author Contributions

HP and WL conceived the research project. FZ, YC, and HZ performed the experiments. WL, XL, and SR performed the data analysis. HP, WL, RH, SJM, XL and JJ interpret the data and discussed the results. WL wrote the manuscript. All authors contributed to the article and approved the submitted version.

## Acknowledgments

This work was sponsored by grants from Shenzhen Science and Technology Project (JCYJ20190813104207152 and JCYJ20170818111629778), NSFC projects (Nos. 32073015, 31670129, and 31802343), Southern Marine Science and Engineering Guangdong Laboratory (Zhan jiang) (No. ZJW-2019-06). The author SR thankfully acknowledges China Post-doctoral Science Foundation for the financial support to carry out the Post-doctoral research work (Grant No. 2019M662214). We also thank Novogene Co., Ltd. for the technical support.

## Conflict of Interest

The authors declare that the research was conducted in the absence of any commercial or financial relationships that could be construed as a potential conflict of interest.

## Supplementary Material

Supplementary Table 1 The identified succinylated proteins and sites in *V. alginolyticus*.

Supplementary Table 2 The identified succinylated peptides in *V. alginolyticus*.

## References

[B1] Avison M. B. Bennett P. M. Howe R. A. Walsh T. R. (2002). Preliminary analysis of the genetic basis for vancomycin resistance in *Staphylococcus aureus* strain Mu50. J. Antimicrob. Chemother. 49, 255–260. 10.1093/jac/49.2.255 11815565

[B2] Avsever M. L. (2016). First report of *trh* positive *Vibrio alginolyticus* isolates from bivalve molluscs in Turkey. Rev. Med. Vet. 167, 65–70.

[B3] Cai S. H. Wu Z. H. Jian J. C. Lu Y. S. (2007). Cloning and expression of gene encoding the thermostable direct hemolysin from *Vibrio alginolyticus* strain HY9901, the causative agent of vibriosis of crimson snapper (*Lutjanus erythopterus*). J. Appl. Microbiol. 103, 289–296. 10.1111/j.1365-2672.2006.03250.x 17650188

[B4] Chatzi K. E. Sardis M. F. Karamanou S. Economou A. (2013). Breaking on through to the other side: protein export through the bacterial Sec system. Biochem. J. 449, 25–37. 10.1042/BJ20121227 23216251

[B5] Chen Y. Y. Wu F. L. Pang H. Y. Tang J. F. Cai S. Cai S. H. (2019a). Superoxide dismutase B (*sodB*), an important virulence factor of *Vibrio alginolyticus*, contributes to antioxidative stress and its potential application for live attenuated vaccine. Fish Shellfish Immunol. 89, 354–360. 10.1016/j.fsi.2019.03.061 30959182

[B6] Chen Y. Y. Wu F. L. Wang Z. W. Tang J. F. Cai S. H. Jian J. C. (2019b). Construction and evaluation of *Vibrio alginolyticus ΔclpP* mutant, as a safe live attenuated vibriosis vaccine. Fish Shellfish Immunol. 98, 917–922. 10.1016/j.fsi.2019.11.054 31770644

[B7] Cheng A. Grant C. E. Bailey T. L. Noble W. S. (2017). MoMo: Discovery of post-translational modification motifs. BioRxiv 153882. 10.1101/153882 PMC6691336 30596994

[B8] Cheng Z. X. Guo C. Chen Z. G. Yang T. C. Zhang J. Y. Wang J. (2019). Glycine, serine and threonine metabolism confounds efficacy of complement-mediated killing. Nat. Commun. 10, 3325. 10.1038/s41467-019-11129-5 31346171 PMC6658569

[B9] Colak G. Xie Z. Y. Zhu A. Y. Dai L. Z. Lu Z. K. Zhang Y. (2013). Identification of lysine succinylation substrates and the succinylation regulatory enzyme CobB in *Escherichia coli* . Mol. Cell. Proteomics 12, 3509–3520. 10.1074/mcp.M113.031567 24176774 PMC3861704

[B10] Coulthurst S. J. Kurz C. L. Salmond G. P. C. (2004). *luxS* mutants of *Serratia* defective in autoinducer-2-dependent ‘quorum sensing’ show strain-dependent impacts on virulence and production of carbapenem and prodigiosin. Microbiology 150, 1901–1910. 10.1099/mic.0.26946-0 15184576

[B11] Crane J. M. Randall L. L. (2017). The Sec system: protein export in *Escherichia coli* . Ecosal Plus 7:10. 10.1128/ecosalplus.ESP-0002-2017 PMC5807066 29165233

[B12] Dan G. Zhang J. Hao Y. Xu R. J. Zhang Y. X. Ma Y. (2019). Alternative sigma factor RpoX is a part of RpoE regulon and plays distinct roles in stress response, motility, biofilm formation and hemolytic activities in the marine pathogen *Vibrio alginolyticus* . Appl. Environ. Microbiol. 85, e00234–e00219. 10.1128/AEM.00234-19 31053580 PMC6606886

[B13] Echazarreta M. A. Klose K. E. (2019). *Vibrio* flagellar synthesis. Front. Cell. Infect. Microbiol. 9, 131. 10.3389/fcimb.2019.00131 31119103 PMC6504787

[B14] Erhardt M. (2016). Strategies to block bacterial pathogenesis by interference with motility and chemotaxis. Curr. Top. Microbiol. Immunol. 398, 185–205. 10.1007/82_2016_493 27000091

[B15] Feng S. G. Jiao K. L. Guo H. Jiang M. Y. Hao J. Wang H. Z. (2017). Succinyl-proteome profiling of *Dendrobium officinale*, an important traditional Chinese orchid herb, revealed involvement of succinylation in the glycolysis pathway. BMC Genomics 18, 598. 10.1186/s12864-017-3978-x 28797234 PMC5553593

[B16] Ferrini A. M. Mannoni V. Suffredini E. Cozzi L. Croci L. (2008). Evaluation of antibacterial resistance in *Vibrio* strains isolated from imported seafood and Italian aquaculture settings. Food Anal. Methods 1, 164–170. 10.1007/s12161-007-9011-2

[B17] Gaviard C. Broutin I. Cosette P. De E. Jouenne T. Hardouin J. (2018). Lysine succinylation and acetylation in *Pseudomonas aeruginosa* . J. Proteome Res. 17, 2449–2459. 10.1021/acs.jproteome.8b00210 29770699

[B18] Gaviard C. Cosette P. Jouenne T. Hardouin J. (2019). LasB and CbpD virulence factors of *Pseudomonas aeruginosa* carry multiple post-translational modifications on their lysine residues. J. Proteome Res. 18, 923–933. 10.1021/acs.jproteome.8b00556 30672296

[B19] Goldberg M. B. Boyko S. A. Butterton J. R. Stoebner J. A. Calderwood S. B. (2010). Characterization of a *Vibrio cholerae* virulence factor homologous to the family of TonB-dependent proteins. Mol. Microbiol. 6, 2407–2418. 10.1111/j.1365-2958.1992.tb01415.x 1406279

[B20] Green E. R. Mecsas J. (2016). Bacterial secretion systems: an overview. Microbiol. Spectr. 4, 213–239. 10.1128/microbiolspec.VMBF-0012-2015 PMC4804464 26999395

[B21] Guo M. L. Huang Z. W. Yang J. (2017). Is there any crosstalk between the chemotaxis and virulence induction signaling in *Agrobacterium tumefaciens* ? Biotechnol. Adv. 35, 505–511. 10.1016/j.biotechadv.2017.03.008 28342941

[B22] He D. L. Wang Q. Li M. Damaris R. N. Yi X. L. Cheng Z. Y. (2016). Global proteome analyses of lysine acetylation and succinylation reveal the widespread involvement of both modification in metabolism in the embryo of germinating rice seed. J. Proteome Res. 15, 879–890. 10.1021/acs.jproteome.5b00805 26767346

[B23] Hernández-Robles M. F. lvarez-Contreras A. K. Juárez-García P. Natividad-Bonifacio I. Curiel-Quesada E. Vázquez-Salinas C. (2016). Virulence factors and antimicrobial resistance in environmental strains of *Vibrio alginolyticus* . Int. Microbiol. 19, 191–198. 10.2436/20.1501.01.277 28504816

[B24] Horii T. Morita M. Muramatsu H. Monji A. Miyagishima D. Kanno T. (2005). Antibiotic resistance in *Aeromonas hydrophila* and *vibrio alginolyticus* isolated from a wound infection: a case report. J. Trauma 58, 196–200. 10.1097/01.ta.0000066381.33339.c0 15674175

[B25] Huang L. X. Xu W. Su Y. Q. Zhao L. M. Yan Q. P. (2018). Regulatory role of the RstB-RstA system in adhesion, biofilm production, motility, and hemolysis. MicrobiologyOpen 7, e00599. 10.1002/mbo3.599 29573209 PMC6182747

[B26] Jin W. B. Wu F. L. (2016). Proteome-wide identification of lysine succinylation in the proteins of Tomato (*Solanum lycopersicum*). PloS One 11, e0147586. 10.1371/journal.pone.0147586 26828863 PMC4734689

[B27] Kapitein N. Mogk A. (2013). Deadly syringes: type VI secretion system activities in pathogenicity and interbacterial competition. Curr. Opin. Microbiol. 16, 52–58. 10.1016/j.mib.2012.11.009 23290191

[B28] Komine-Abe A. Nagano-Shoji M. Kubo S. Kawasaki H. Yoshida M. Nishiyama M. (2017). Effect of lysine succinylation on the regulation of 2-oxoglutarate dehydrogenase inhibitor, OdhI, involved in glutamate production in *Corynebacterium glutamicum* . Biosci. Biotechnol. Biochem. 81, 2130–2138. 10.1080/09168451.2017.1372182 28899215

[B29] Kosono S. Tamura M. Suzuki S. Kawamura Y. Yoshida A. Nishiyama M. (2015). Changes in the acetylome and succinylome of *Bacillus subtilis* in response to carbon source. PloS One 10, e0131169. 10.1371/journal.pone.0131169 26098117 PMC4476798

[B30] Li X. L. Hu X. Wan Y. J. Xie G. Z. Li X. Z. Chen D. (2014). Systematic identification of the lysine succinylation in the protozoan parasite *Toxoplasma gondii* . J. Proteome Res. 13, 6087–6095. 10.1021/pr500992r 25377623

[B31] Nadine P. Serge G. Yang X. J. (2017). Assays for acetylation and other acylations of lysine residues. Curr. Protoc. Protein Sci. 87, 1–18. 10.1002/cpps.26 28150880

[B32] Pan J. Y. Chen R. Li C. C. Li W. Y. Ye Z. C. (2015). Global analysis of protein lysine succinylation profiles and their overlap with lysine acetylation in the marine bacterium *Vibrio parahemolyticus* . J. Proteome Res. 14, 4309–4318. 10.1021/acs.jproteome.5b00485 26369940

[B33] Pang H. Y. Li W. X. Zhang W. J. Zhou S. H. Hoare R. Monaghan S. J. (2020). Acetylome profiling of *Vibrio alginolyticus* reveals its role in bacterial virulence. J. Proteomics 211, 103543. 10.1016/j.jprot.2019.103543 31669173

[B34] Ren J. Sang Y. Lu J. Yao Y. F. (2017). Protein acetylation and its role in bacterial virulence. Trends Microbiol. 25, 768–779. 10.1016/j.tim.2017.04.001 28462789

[B35] Ribet D. Cossart P. (2010). Pathogen-mediated posttranslational modifications: a re-emerging field. Cell 143, 694–702. 10.1016/j.cell.2010.11.019 21111231 PMC7112265

[B36] Samuelson J. C. Chen M. Y. Jiang F. L. Möller I. Dalbey R. E. (2000). YidC mediates membrane protein insertion in bacteria. Nature 406, 637–641. 10.1038/35020586 10949305

[B37] Santhakumari S. Nilofernisha N. M. Ponraj J. G. Pandian S. K. Ravi A. V. (2017). In vitro and in vivo exploration of palmitic acid from *Synechococcus elongatus* as an antibiofilm agent on the survival of Artemia franciscana against virulent vibrios. J. Invertebr. Pathol. 150, 21–31. 10.1016/j.jip.2017.09.001 28887169

[B38] Sasikala D. Srinivasan P. (2016). Characterization of potential lytic bacteriophage against *Vibrio alginolyticus* and its therapeutic implications on biofilm dispersal. Microb. Pathog. 101, 24–35. 10.1016/j.micpath.2016.10.017 27793690

[B39] Song Y. X. Wang J. Cheng Z. Y. Gao P. Sun J. X. Chen X. W. (2017). Quantitative global proteome and lysine succinylome analyses provide insights into metabolic regulation and lymph node metastasis in gastric cancer. Sci. Rep. 7, 42053. 10.1038/srep42053 28165029 PMC5292683

[B40] Sun L. N. Yao Z. J. Guo Z. Zhang L. S. Wang Y. Q. Mao R. R. (2019). Comprehensive analysis of the lysine acetylome in *Aeromonas hydrophila* reveals cross-talk between lysine acetylation and succinylation in LuxS. Emerg. Microbes Infect. 8, 1229–1239. 10.1080/22221751.2019.1656549 31448697 PMC6735345

[B41] Wang Q. Y. Liu Q. Cao X. D. Yang M. J. Zhang Y. X. (2008). Characterization of two TonB systems in marine fish pathogen *Vibrio alginolyticus*: their roles in iron utilization and virulence. Arch. Microbiol. 190, 595–603. 10.1007/s00203-008-0407-1 18629473

[B42] Wang Y. Q. Wang X. Y. Ali F. Li Z. Q. Fu Y. Y. Yang X. J. (2019). Comparative extracellular proteomics of *Aeromonas hydrophila* reveals iron-regulated secreted proteins as potential vaccine candidates. Front. Immunol. 10, 256. 10.3389/fimmu.2019.00256 30833947 PMC6387970

[B43] Weinert B. T. Schölz C. Wagner S. A. Iesmantavicius V. Su D. Daniel J. A. (2013). Lysine succinylation is a frequently occurring modification in prokaryotes and eukaryotes and extensively overlaps with acetylation. Cell Rep. 4, 842–851. 10.1016/j.celrep.2013.07.024 23954790

[B44] Whitmore S. E. Lamont R. J. (2012). Tyrosine phosphorylation and bacterial virulence. Int. J. Oral. Sci. 4, 1–6. 10.1038/ijos.2012.6 22388693 PMC3412661

[B45] Wu L. Gong T. Zhou X. D. Zeng J. M. Huang R. J. Wu Y. F. (2019). Global analysis of lysine succinylome in the periodontal pathogen *Porphyromonas gingivalis* . Mol. Oral. Microbiol. 34, 74–83. 10.1111/omi.12255 30672658

[B47] Xie L. X. Liu W. Li Q. M. Chen S. D. Xu M. M. Huang Q. Q. (2014). First succinyl-proteome profiling of extensively drug-resistant *Mycobacterium tuberculosis* revealed involvement of succinylation in cellular physiology. J. Proteome Res. 14, 107–109. 10.1021/pr500859a 25363132

[B46] Xie L. X. Li J. Deng W. Y. Yu Z. X. Fang W. J. Chen M. (2017). Proteomic analysis of lysine succinylation of the human pathogen *Histoplasma capsulatum* . J. Proteomics 154, 109–117. 10.1016/j.jprot.2016.12.020 28063982

[B48] Xiong X. P. Wang C. Ye M. Z. Yang T. C. Peng X. X. Li H. (2010). Differentially expressed outer membrane proteins of *Vibrio alginolyticus* in response to six types of antibiotics. Mar. Biotechnol. 12, 686–695. 10.1007/s10126-009-9256-4 20217167

[B49] Xu H. Chen X. Y. Xu X. L. Shi R. Y. Suo S. S. Cheng K. Y. (2016). Lysine acetylation and succinylation in HeLa cells and their essential roles in response to UV-induced stress. Sci. Rep. 6, 30212. 10.1038/srep30212 27452117 PMC4959001

[B50] Yang M. K. Wang Y. Chen Y. Cheng Z. Y. Gu J. Deng J. Y. (2015). Succinylome analysis reveals the involvement of lysine succinylation in metabolism in pathogenic *Mycobacterium tuberculosis* . Mol. Cell. Proteomics 14, 796–811. 10.1074/mcp.M114.045922 25605462 PMC4390261

[B51] Yang X. J. Seto E. (2008). Lysine acetylation: codified crosstalk with other posttranslational modifications. Mol. Cell 31, 449–461. 10.1016/j.molcel.2008.07.002 18722172 PMC2551738

[B52] Yao Z. J. Guo Z. Wang Y. Q. Li W. X. Fu Y. Y. Lin Y. X. (2019). Integrated succinylome and metabolome profiling reveals crucial role of S-ribosylhomocysteine lyase in quorum sensing and metabolism of *Aeromonas hydrophila* . Mol. Cell. Proteomics 18, 200–215. 10.1074/mcp.RA118.001035 30352804 PMC6356075

[B53] Yu Q. Liu M. Z. Su H. F. Xiao H. H. Wu S. T. Qin X. L. (2019). Selection and characterization of ssDNA aptamers specifically recognizing pathogenic *Vibrio alginolyticus* . J. Fish Dis. 42, 851–858. 10.1111/jfd.12985 30859598

[B55] Zhang Z. H. Tan M. J. Xie Z. Y. Dai L. Z. Chen Y. Zhao Y. M. (2011). Identification of lysine succinylation as a new post-translational modification. Nat. Chem. Biol. 7, 58–63. 10.1038/nchembio.495 21151122 PMC3065206

[B54] Zhang Y. M. Wang G. Y. Song L. M. Mu P. Wang S. Liang W. X. (2017). Global analysis of protein lysine succinylation profiles in common wheat. BMC Genomics 18, 1–10. 10.1186/s12864-017-3698-2 28427325 PMC5397794

[B56] Zheng H. L. He Y. Zhou X. W. Qian G. Y. Lv G. X. Shen Y. N. (2016). Systematic analysis of the lysine succinylome in *Candida albicans* . J. Proteome Res. 15, 3793–3801. 10.1021/acs.jproteome.6b00578 27605073

[B57] Zheng Z. W. Ye L. W. Chan E. W. C. Chen S. (2019). Identification and characterization of a conjugative bla VIM-1-bearing plasmid in *Vibrio alginolyticus* of food origin. J. Antimicrob. Chemother. 74, 1842–1847. 10.1093/jac/dkz140 30993329

[B59] Zhou H. Finkemeier I. Guan W. X. Tossounian M. Wei B. Young D. (2018). Oxidative stress-triggered interactions between the succinyl- and acetyl-proteomes of rice leaves. Plant Cell Environ. 41, 1139–1153. 10.1111/pce.13100 29126343

[B58] Zhou C. L. Dai J. L. Lu H. Z. Chen Z. J. Guo M. He Y. (2019). Succinylome analysis reveals the involvement of lysine succinylation in the extreme resistance of *Deinococcus radiodurans* . Proteomics 19, e1900158. 10.1002/pmic.201900158 31487437

